# Periodontitis, Microbiomes and their Role in Alzheimer’s Disease

**DOI:** 10.3389/fnagi.2017.00336

**Published:** 2017-10-24

**Authors:** Anna B. Pritchard, StJohn Crean, Ingar Olsen, Sim K. Singhrao

**Affiliations:** ^1^Dementia & Neurodegenerative Diseases Research Group, Faculty of Clinical and Biomedical Sciences, School of Dentistry, University of Central Lancashire, Preston, United Kingdom; ^2^Department of Oral Biology, Faculty of Dentistry, University of Oslo, Oslo, Norway

**Keywords:** Alzheimer’s disease, infections, functional amyloids, microbiomes, periodontitis

## Abstract

As far back as the eighteenth and early nineteenth centuries, microbial infections were responsible for vast numbers of deaths. The trend reversed with the introduction of antibiotics coinciding with longer life. Increased life expectancy however, accompanied the emergence of age related chronic inflammatory states including the sporadic form of Alzheimer’s disease (AD). Taken together, the true challenge of retaining health into later years of life now appears to lie in delaying and/or preventing the progression of chronic inflammatory diseases, through identifying and influencing modifiable risk factors. Diverse pathogens, including periodontal bacteria have been associated with AD brains. Amyloid-beta (Aβ) hallmark protein of AD may be a consequence of infection, called upon due to its antimicrobial properties. Up to this moment in time, a lack of understanding and knowledge of a microbiome associated with AD brain has ensured that the role pathogens may play in this neurodegenerative disease remains unresolved. The oral microbiome embraces a range of diverse bacterial phylotypes, which especially in vulnerable individuals, will excite and perpetuate a range of inflammatory conditions, to a wide range of extra-oral body tissues and organs specific to their developing pathophysiology, including the brain. This offers the tantalizing opportunity that by controlling the oral-specific microbiome; clinicians may treat or prevent a range of chronic inflammatory diseases orally. Evolution has equipped the human host to combat infection/disease by providing an immune system, but *Porphyromonas gingivalis* and selective spirochetes, have developed immune avoidance strategies threatening the host-microbe homeostasis. It is clear from longitudinal monitoring of patients that chronic periodontitis contributes to declining cognition. The aim here is to discuss the contribution from opportunistic pathogens of the periodontal microbiome, and highlight the challenges, the host faces, when dealing with unresolvable oral infections that may lead to clinical manifestations that are characteristic for AD.

## Introduction

Alzheimer’s disease (AD) is the most common example of dementia causing around 60%–80% of all cases (Gaugler et al., 2016). AD is characterized by cognitive deficit and has a complex, multifactorial etiology. The limited treatment options make it a challenging condition in which neuropsychiatrists/neurologists can do little to help their patients. The relentlessly downward course of the disease has impact on both the patient and their carers. Furthermore, the rising aging population and the predicted increase in the prevalence of the disease has immediate and long-term socioeconomic implications (Prince et al., 2014). Classically there are two forms of this neurodegenerative condition. The familial/early-onset form displaying an earlier manifestation, albeit in fewer (<5%) AD cases is overall, more severe with increased functional loss and Aβ deposits (Bekris et al., 2010). Susceptibility genes and their co-expressing environmental factors appear to exert influence over the sporadic/late-onset form, which accounts for the majority (95%) of AD cases. AD has a bi-phasic criterion of diagnosis, which involves correlation of the clinical presentation with neuropathological examination, at post-mortem (Braak and Braak, 1995). Despite the difference between familial and sporadic AD, the underlying neuropathology remains common to both forms.

The two diagnostic neuropathological hallmarks are numerous extracellular deposits of amyloid-beta (Aβ plaques) and neurofibrillary tangles (NFTs) in the frontal cortex and the hippocampal areas of the brain (Braak and Braak, 1995). In the brain, Aβ can take different physiological states (soluble monomers, dimers, insoluble Aβ_40/42_), which eventually result in an insoluble, stable β-helical sheet structure in the form of two morphologically different plaques. The senile plaques are composed of densely packed Aβ_1–42_, and known for their cytotoxicity in causing demise of surrounding neurons (McGeer et al., 2017). The NFTs, located within cortical neurons, are composed of paired helical filaments (PHFs) and tau protein, the latter of which undergoes posttranslational modification in the form of hyperphosphorylation.

In addition to the classic diagnostic hallmark proteins, is the apparently asymptomatic developing neuropathology. Biomarker studies using positron emission tomography for levels of Aβ, and magnetic resonance imaging for brain volume, indicate that AD onset begins years before the clinical picture emerges (McGeer et al., 2017). Cerebral inflammation in the form of activated glia (microglia and astrocytes; Norden and Godbout, 2013), and Aβ_1–40_ plaques are two of the many asymptomatic features seen in AD. This implies that age-related priming of glia due to bacterial entry from hosts’ dysbiotic microbiomes elsewhere in the body (oral, gastrointestinal (GI) tract) provide slow inflammatory damage. In support, oral pathogens, especially *Porphyromonas gingivalis*, thrive under toxic inflammatory conditions and if present, may dampen the early pathogenic effects of glial cell activation (Singhrao et al., 2015). This makes the findings of a recent retrospective cohort study highlighting a 10-year exposure to chronic periodontitis may lead to manifesting AD (Chen et al., 2017), very plausible. Whether inflammation precedes Aβ or vice versa, is not clear; but considering Aβ as an innate immune protein with its antimicrobial properties (Soscia et al., 2010; Kumar et al., 2016) would place infections upstream of this hallmark protein. A suggested sequence of AD pathophysiology would likely follow infections, Aβ deposition and glial cell activation. These three events may also provide an explanation for the age related glial cell priming by genetic switches turning on, during life, in an age dependent manner (Jayadev et al., 2013; Fan et al., 2017; Wu et al., 2017).

**Figure 1:**
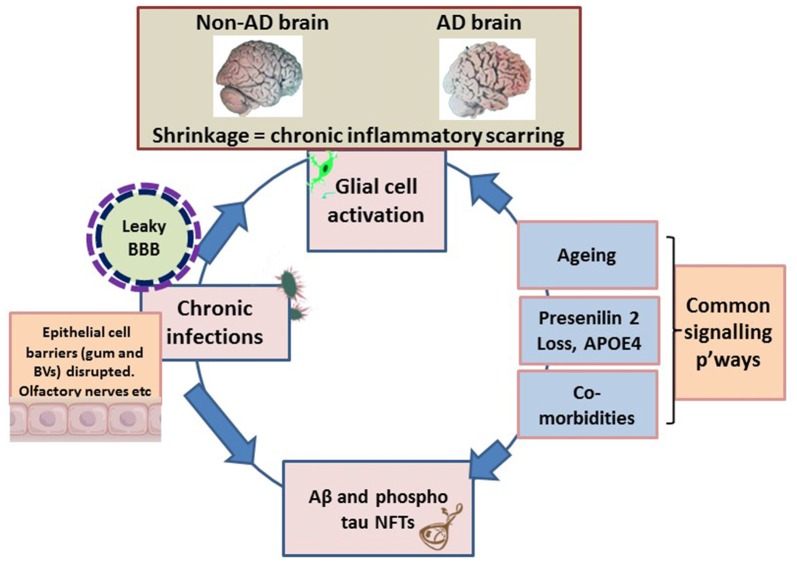
Schematic illustrating the macroscopic features relating to shrinkage (wider sulci, compared with non-Alzheimer’s disease (AD) brain), unique to the AD brain, which equates to inflammatory condition. The pathogens disrupt the epithelial cell-to-cell proteins of the gingivae through their proteases. The epithelial/endothelial barriers of capillaries disrupted for effective bacteremia to take place. The olfactory nerve pathways exploited to evade immune recognition. Environmental factors are the inflammophilic microbes with potential to subvert hosts immune defenses that also contribute to common inflammatory activities/pathways (p’ways) as well as contributing to proteostasis. At this stage the brain’s resilience is markedly compromised and the blood-brain barrier (BBB) is becoming defective. The endotoxin intolerance/further inflammation tip the brain into disease.

If atrophy of soft and bony tissues classically refers to decreased cell size/cell loss, such an observation can equally, imply shrinkage as a marker post inflammation. Brain atrophy is also a feature of multiple sclerosis (Pérez-Cerdá et al., 2016), which potentially presents with similar immune (innate and adaptive) tissue response as that seen in periodontal disease (Di Benedetto et al., 2013; Olsen et al., 2016). In periodontitis, the localized bacterial accumulation results in stimulations that elicit inflammation and activation of the innate immune system. Influx of systemic inflammatory cells follows a short time afterwards by an adaptive immune cell response leading to tissue loss (Di Benedetto et al., 2013; Olsen and Singhrao, 2015; Olsen et al., 2016). Recent evidence suggests that the adaptive immune system may have an important role in suppressing AD neuropathology (Marsh et al., 2016; Olsen et al., 2016). It may therefore be that, with aging and a waning adaptive immune system, AD neuropathology may be more likely to be evident. Additional to this, both periodontal pathogens (*P. gingivalis* and *Treponema denticola*) show weak responses for attracting systemic inflammatory cells (neutrophils, T/B cells) into the brain (Olsen et al., 2016). However, the concept of inflammation and macroscopic atrophic appearance (enlarged sulci and ventricles) unique to AD brains may offer similar clues for a pivotal and primary role of inflammation (Figure 1) at the organ level.

The atrophic appearance of AD brains corroborates inflammation and is a compelling indication of numerous bacteria/bacterial endo/exo/toxins and fungi/viruses observed in association with Aβ plaques (Hill et al., 2014; Itzhaki, 2016; Lukiw, 2016; Pistollato et al., 2016; Alonso et al., 2017; Harris and Harris, 2017; Jiang et al., 2017; Maheshwari and Eslick, 2017; Zhao et al., 2017). The large microbial biodiversity identified from post-mortem AD brain specimens could be because of the differences in age, diet, lifestyle, geographical environment and disease status, a limitation also recognized by the human microbiome project^1^. This places a greater onus on microbial virulence factor(s)/pathogen associated molecular patterns (PAMPs) than live microbes exerting a pathological effect with the common end-point of this neurodegenerative disease. An example of this is the detection of lipopolysaccharide (LPS) in AD brains with the resulting opsonization of LPS-producing bacteria by glial cells (Poole et al., 2013), and their direct binding with Aβ plaques (Zhan et al., 2016; Zhao et al., 2017). Undoubtedly, LPS from the outer membrane of Gram-negative bacteria is a powerful pro-inflammatory PAMP. This may carry with it proteolytic enzymes (gingipains, peptidyl deiminases and carbonic anhydrases) and appendages such as fimbriae and curli fibers (curli are functional amyloids housed on the outer membrane of several prokaryotes) and other amyloid-like proteins (Table 1). *In vivo* experimental models have suggested LPS from oral, Gram negative bacteria having a role in chronic local inflammation (DiCarlo et al., 2001); Aβ release (Sheng et al., 2003; Wu et al., 2017); worsened cognition (Wu et al., 2017); and tau protein phosphorylation (Lee et al., 2010). More pathogenic bacteria from the GI tract microbiome appear with curli fibers than presently known for the oral microbiome and therefore show direct associations with plaques (Lukiw, 2016; Pistollato et al., 2016; Zhan et al., 2016; Jiang et al., 2017; Zhao et al., 2017). A recent systematic review in which meta-analysis of periodontitis with AD was conducted, demonstrated a significant association between these two diseases (Odds Ratio (OR) 1.69, 95% CI 1.21–2.35) and an even more significant association was observed in severe form of PD with AD (OR 2.98, 95% CI 1.58–5.62; Leira et al., 2017). A population based retrospective study by Chen et al. (2017) demonstrated a 10 year, exposure of chronic periodontitis led to a higher risk (1.707-fold increase) of developing AD. Our aim here is to understand how the oral microbiome pathogens contribute to the hallmark proteins especially Aβ of AD.

**Table 1:** Prokaryotes and eukaryotes found in biofilm communities with genes for expressing functional amyloids.

Species/Genera/Phylotypes	Protein	Protein function	References
*Pseudomonas*	FapC	Biofilm formation, virulence factor	Larsen et al. (2007) and Dueholm et al. (2010)
*Escherichia coli*	CsgA, Curli	Biofilm formation, virulence factor	Chapman et al. (2002), Dueholm et al. (2012) and Zhan et al. (2016)
*Klebsiella pneumoniae*	Microcin E492	Cytotoxicity	Bieler et al. (2005)
*Mycobacterium tuberculosis*	MTP	Adhesive pili	Alteri et al. (2007)
*Bacillus subtilis*	TasA	Biofilm	Romero et al. (2010, 2011, 2014)
*Salmonella enterica*	Curli	Biofilm, virulence factor	Solomon et al. (2005)
*Salmonella typhimurium*	Curli	Biofilm, virulence factor	Castelijn et al. (2012)
*Streptococcus mutans*	Adhesin P1	Biofilm, caries	Oli et al. (2012)
Bacteroidetes	Curli	Biofilm	Larsen et al. (2008) and Dueholm et al. (2012)
Chloroflexi	Curli	Biofilm	Larsen et al. (2008) and Dueholm et al. (2012)
Firmicutes	Curli	Biofilm	Dueholm et al. (2012)
Thermodesulfobacteria	Curli	Biofilm	Dueholm et al. (2012)
Alpha, beta, delta and gamma-proteoabacteria	Curli	Biofilm	Larsen et al. (2007, 2008), Dueholm et al. (2012, 2013),
*Candida albicans*	Als	Adhesion	Ramsook et al. (2010) and Garcia et al. (2013)

## The Oral Microbiome and Periodontitis

Periodontal disease is one of the most common chronic polymicrobial infections in humans, characterized by loss of tooth supporting tissues due to the host’s immuno-inflammatory responses, and consequently are a major cause of loss of teeth. Loss of more than 16 teeth in early to mid-life is significantly associated with the development of dementia with OR of 1.56 (95% CI 1.12–2.18; Gatz et al., 2006; Luo et al., 2015). Conversely, retaining teeth and not brushing them, also exposes individuals to the risk of developing dementia. Taken together, the plausible explanation for missing and unclean teeth is poor oral hygiene (Paganini-Hill et al., 2012). Periodontitis is prevalent in individuals with poor oral hygiene and high dental plaque index.

The oral cavity is a home to over 700 different taxa and undoubtedly, this specific microbiome^2^ will keep growing, as changing bacterial species are included. At present, much smaller numbers 1 × 10^9^/pocket of mixed bacterial phyla (Loesche and Lopatin, 1998) are associated with periodontitis within this subgingival niche compared to the oral cavity where 1 × 10^11^ bacteria/mg of dental plaque are recorded (Li et al., 2000). Dental plaque is a biofilm of a synergistic microbial community, and both genetic and environmental factors can cause it to become dysbiotic and lead to clinical manifestations of periodontitis. Some common examples of bacteria in the periodontal microbiome include *P. gingivalis, Tannerella forsythia, Prevotella intermedia, Eikenella corrodens, Fusobacterium nucleatum, Aggregatibacter actinomycetemcomitans* and *T. denticola*^2^. The Gram-negative bacterium *P. gingivalis*, is a keystone pathogen that modulates the dysbiosis of its companion species of bacteria beneath the gingivae (Hajishengallis and Lamont, 2014). The dysbiotic microbial community model of periodontal disease explains how a wide range and changing phyla can participate in generating pathology under the influence of this keystone pathogen (Hajishengallis and Lamont, 2016). The chronicity of periodontal disease, resulting in a potentially prolonged assault by a group of pathogens on the host’s system, opens up the opportunity of specific microbes inducing a state where the host’s threshold for disease exceeds that of health allowing local and remote organ pathology to develop.

## Oral Microbiomes Contribution to AD

Previous reports have alluded to the existence of an unexplored microbiome in the elderly and AD brains (Riviere et al., 2002), but the age at which infection takes hold in the brain is unknown. Microbiomes play an important role in balancing health and disease boundaries. Microbes (oral and non-oral) are implicated in the etiology of AD; this includes *Borrelia* species, *T. denticola, P. gingivalis* and *Escherichia coli* (Miklossy, 2011; Poole et al., 2013; Zhan et al., 2016), oral fungi (Carrasco et al., 2017) and others (Olsen and Singhrao, 2015; Maheshwari and Eslick, 2017). The diversity of microbes documented could be a reflection of the brain donor’s geographical location, their age, diet, oral function (denture wearing) and lifestyle (Yatsunenko et al., 2012; Lukiw, 2013; Danborg et al., 2014; Heintz and Mair, 2014). Several scientists including us believe that the AD brain harbors its own microbiome (Emery et al., 2017), which may be due to the contribution of “radicalized” bacteria (Harding et al., 2017) from other human microbiomes (mouth, skin, GI tract), and as a consequence of the food chain and co-morbid states (Singhrao et al., 2016). Since age is the major risk factor for developing AD, then evolving microbiomes may provide dynamics of the microbial communities over time. To this end, Brandscheid et al. (2017) examined changes in microbial communities in the GI tract of the 5XFAD AD transgenic mouse model and confirmed changes to microbial communities occurred over time. The changing microbes correlated with changes in trypsin secretions (Brandscheid et al., 2017), implying meat rich diets are indigestible in old age and excess protein may upset the existing microbial community dynamics. If two main phyla of GI tract bacteria are Firmicutes (approximately 80%) and Bacteroidetes (approximately 20%; Lukiw, 2016); it appears that during aging humans also undergo shifts in favor of Bacteroidetes in their GI tract microbiome (Pistollato et al., 2016). *P. gingivalis* is a species within the genus *Bacteroides* (within the phylum Bacteroidetes) and with periodontitis becoming more chronic and prevalent in old age, this would imply that the periodontal microbiome offers a relatively early indication of changing microbial dynamics.

An AD-specific microbiome might be composed of bacteria from associated dysbiotic microbiomes because microbial infections explain the common inflammatory pathways (Olsen and Singhrao, 2015; Lukiw, 2016; Olsen et al., 2016) and their effects in the elderly brain via host’s peripheral immune responses and related signaling pathways (see “LPS in AD Inflammatory Cascades” section). Undoubtedly, a complex etiology underlies the clinical manifestations seen in AD giving rise to new concepts. It is time to re-examine the infection model that asks: are microbes the causative agents of AD? This pertinent question anxiously awaits answers (Fischer, 1910; MacDonald and Miranda, 1987; Miklossy, 1993; Mawanda and Wallace, 2013; Olsen and Singhrao, 2015; Itzhaki et al., 2016). To this end, the biofilm concept of AD senile plaques is proposed (Allen et al., 2016; Miklossy, 2016) and offers an alternative platform for answering this fundamental question.

## Functional Amyloids from Prokaryotic Origins

The prokaryotic functional amyloid (Epstein and Chapman, 2008; Dueholm et al., 2013), affords diverse functions in biofilm communities that range from structural components namely, fimbriae, curli and other cellular appendages to act as oligomeric toxins (Larsen et al., 2007; Dueholm et al., 2013); reservoirs for quorum sensing; signaling molecules and binding of redox mediators (Dueholm and Nielsen, 2017). Furthermore, there is potential for these prokaryotic Aβ-like fibers to cross the blood-brain barrier (BBB) and form pathological senile plaques seen in AD. Presence of curli protein is emphasized by the GI tract bacteria (Pistollato et al., 2016; Jiang et al., 2017; Zhao et al., 2017) and as an analogy to prion plaques, help to explain the protein-protein interactions leading to senile plaque formation connecting the biofilm hypothesis (Allen et al., 2016; Miklossy, 2016).

Shahnawaz and Soto (2012), reported that the functional amyloid MccE492 from *Klebsiella pneumoniae* RYC492 has the ability to depolymerize from its fibrillary state and release oligomers capable of inducing cytotoxicity equivalent to pathological Aβ in AD. This paved the way for the molecular mimicry theory (Hartman et al., 2013; Friedland, 2015). The molecular mimicry theory incorporates microbial curli fibers and human Aβ as a protein-protein interaction, which can result in cross-seeding even if these proteins are dissimilar (Friedland, 2015). Additional prokaryotic and yeast functional amyloid systems and their sources are listed in Table 1 (Bian et al., 2000; Gophna et al., 2001; Larsen et al., 2007, 2008; Dueholm et al., 2010, 2012, 2013; Dueholm and Nielsen, 2017).

Infections induce acute phase proteins as part of the innate immune response (Gabay and Kushner, 1999). One of these is serum amyloid component P (SAP). SAP can bind *Candida albicans* (Klotz et al., 2016) in the similar way Aβ binds to bacteria in its antimicrobial peptide capacity (Soscia et al., 2010; Kumar et al., 2016). This implies that amyloid-like proteins (Table 1) from foreign and host sources undergo physical protein-protein interactions with potential to cross-seed with Aβ, and increase the amyloid burden. These extrinsic proteins (curli) are PAMPs (Bian et al., 2000) with β-pleated sheet structures (Friedland, 2015), and they cross-react with antibodies to human Aβ plaques (Miklossy, 2016). Due to their density and morphological appearance, the senile plaques may be composed of curli-like Aβ_1–42_ compositions. Would it then be plausible to suggest that either AD is a result of mixed pathologies or it has multiple etiological agents (Figure 1) that bypass protective host barriers, whereby some give rise to Aβ_1–40_ and others like curli fibers, cross-seed to generate Aβ_1–42_? The molecular mimicry/Aβ cross-seeding hypothesis for bacterial phylotypes (Hartman et al., 2013; Friedland, 2015) is attractive as it gives microbes a more prominent role in AD causality.

## Senile Plaque, a Miniature Biofilm Hypothesis

The proposal that pathogenic microbes (including oral spirochetes) are able to manifest the pathological and biological hallmarks of AD led Allen et al. (2016) and Miklossy (2016) to propose that “the senile plaques” in syphilitic and Lyme disease brain specimens are conglomerates of pathogenic bacteria, and can be viewed as multispecies biofilms. This compelling notion supports the cross-reactivity of antibodies that detect the breakdown product of the amyloid precursor protein (APP) Aβ in human AD brains, with the “Aβ-like” senile plaques formed by spirochaetal aggregates *in vitro* (Miklossy, 2016). In support of the Allen and Miklossy biofilm hypothesis, Friedland (2015) proposes a mechanism in which curli fibers and/or other similar bacterial antigens, with capacity to aggregate and acquire Aβ conformation would eventually grow by incorporating host Aβ and aggregate in the form of AD senile plaques (Friedland, 2015).

Hundreds of synergistic species of bacteria reside within the organ specific (mouth, skin, GI tract) biofilm ecologies that outnumber the entire cells making up the human body^3^. Given the diversity of microbes identified from AD brains, it is plausible to expect a heterogeneous biofilm in which bacteria, fungi and viruses, all reside side by side. Since it is established that Aβ is an antimicrobial peptide (Soscia et al., 2010), an expected consequence to microbes would be that of death, specifically in the context of AD. This together with the already highly inflamed environment of the AD brain and ongoing glial cell activity is likely to kill bacteria and exclude brain abscesses forming. Therefore, the senile plaques of AD are only likely to retain bacterial virulence factors such as LPS and genomic DNA signatures. Some bacteria and fungi form spores within the AD brain tissue and that way, they bypass the antimicrobial effects of the Aβ_40/42._ Microbiological culture based methodologies allow their detection as previously reported (Balin et al., 2008; Miklossy et al., 2017). This is a testimony to live microbes having entered the AD brain at some stage rather than mere contamination of tissue at post-mortem. *C. albicans* has also been observed in post mortem AD brain specimens (Carrasco et al., 2017) and has functional amyloid-like adhesins (Als) on its cell surface (Ramsook et al., 2010; Garcia et al., 2013). *C. albicans* is an opportunistic yeast found commonly in the oral cavity, where it typically becomes pathogenic in immunosuppressed individuals or if local factors are conducive to its growth. For example, if the individual uses a steroid inhaler or wears dentures (Scully and Felix, 2005). *C. albicans* infection can be asymptomatic and is difficult to eradicate. The concept of trapping/incapacitating and killing bacteria in the brain by Aβ antimicrobial activity (Soscia et al., 2010; Kumar et al., 2016) perhaps equally applies to SAP mediated trapping of *C. albicans* systemically (Klotz et al., 2016). However, this affinity could also be due to the amyloid-like adhesion on the surface of *C. albicans* (Ramsook et al., 2010; Garcia et al., 2013). If the senile plaques represent foci of miniature biofilms, then *C. albicans* may be responsible for adding to the Aβ burden by bringing its own Als (Ramsook et al., 2010; Garcia et al., 2013) and the insoluble SAP from its primary niche (Ramsook et al., 2010; Klotz et al., 2016). As human brain Aβ and bacterial LPS are resistant to degradation, their accumulation is to be expected.

### LPS in AD Proteostasis

The predominant signaling cascades participating in the innate immune system in AD pathogenesis include the Toll-like receptor (TLR) pathways. LPS in rodents demonstrate participation of TLRs, CD14 and NF-κB signaling cascades (Olsen and Singhrao, 2015; Lukiw, 2016) and indicates that acute phase inflammation is beneficial whilst chronic organ specific inflammation is detrimental (DiCarlo et al., 2001; Sheng et al., 2003; Lee et al., 2010; Herber-Jonat et al., 2011) *E. coli* is a member of the oral microbiome^4^. genetically encoded for curli protein, and is included here because of Friedland’s protein-protein interaction hypothesis for its plausible contribution to AD senile plaques (Friedland, 2015). *E. coli* LPS (presumably with curli), also co-localizes with AD senile plaques (Zhan et al., 2016; Zhao et al., 2017). In addition, experiments with peripheral inoculations of LPS from *E. coli* in APPswe transgenic mice (Sheng et al., 2003) have demonstrated increased expression of APP with Aβ release (Sheng et al., 2003). This result supports the concept of peripheral inflammation as an initiating factor in intracerebral inflammatory activity as well as supporting the release of at least one hallmark (Aβ) protein (Sheng et al., 2003). More recently, Wu et al. (2017) demonstrated that repeat injections of LPS from *P. gingivalis*, activated cathepsin B (a form of β secretase) indirectly to cleave APP intracellular fragmentation in an age-dependent manner. Cathepsin B plays a pivotal role in the neuroinflammation induced by *P. gingivalis* LPS followed by intracellular APP cleavage (Wu et al., 2017). Functional testing revealed that chronic and systemic administration of *P. gingivalis* LPS in middle-aged mice caused learning and memory deficits (Wu et al., 2017), supporting an AD-like phenotype and giving this PAMP, from an oral keystone pathogen, a more prominent role in AD causality.

*Salmonella* species enter the oral cavity through consumption of contaminated meat and eggs (Edwards and Bruner, 1938). The importance of *S. abortus equii* (Edwards and Bruner, 1938) lies in its LPS in relation to neuroinflammation and more uniquely in tau protein phosphorylation. *S. abortus equii* LPS inoculations in the hippocampus of rTg4510 mice carrying the parental tau mutations and non-transgenic littermates (Lee et al., 2010), supported an initial neuroinflammatory activity, followed by increased Ser199/202 and phospho-tau Ser396 in the mutated group (Lee et al., 2010). This result demonstrates the role of several bacteria (host microbiome-derived and extrinsic, food chain sources) and hosts’ genetic susceptibility contributing to inflammatory stimuli subsequent to which tau phosphorylation (Lee et al., 2010) takes place. In other words, the signaling pathways participating in innate immune mediator release have the potential to modify the PHF bound tau protein by posttranslational means, in the vulnerable host, at any time. Thus, NFT formation could be both dependent and independent of Aβ deposits.

With relevance to AD pathology, a defective BBB is documented (Montagne et al., 2015; Halliday et al., 2016) and a plausible explanation for the permeability is related to loss of cell-cell tight junctional proteins. LPS from some bacteria such as *P. gingivalis* also contains proteolytic enzymes (gingipains) that cleave and fragment proteins into smaller peptides. The proteolytic activity of gingipains also targets cell-cell adhesion molecules (Katz et al., 2000; Hintermann et al., 2002; Sheets et al., 2005). It is, therefore, likely that gingipains also contribute to the degradation of endothelial cell tight junction proteins, and contribute to loss of BBB functional integrity. In support of this hypothesis, *P. gingivalis* infected animal models demonstrated hippocampal damage via inflammation-mediated injury and IgG and gingipains in the cerebral microvasculature (Singhrao et al., 2017). In addition, the phagocytic oxidative burst of the host’s neutrophils and macrophages at a much earlier time point of *P. gingivalis* infection, and the oxidative stress response initiated by bacteria can equally damage the hippocampal microvasculature (Rokad et al., 2017). A permeable BBB also has implications for entry of extra-cerebral amyloid/amyloid-like proteins to the brain and add to the existing amyloid burden. Furthermore, under appropriate conditions, arginine residues on the end of fragmented proteins can undergo citrullination, a form of posttranslational modification, initiated by *P. gingivalis* peptidyl arginine deiminase (Bielecka et al., 2014). This is particularly relevant for retaining C5a activity and attracting immune cells to the brain (Farkas et al., 2003; Bielecka et al., 2014). This may be why AD pathology lacks presence of systemic phagocytes and T/B cells in AD brains. The insoluble Aβ_40/42_ however does associate with macromolecules such as DNA, LPS, metal ions and other binding proteins (Mueller-Steiner et al., 2006; Itzhaki, 2016; Maher et al., 2016; Zhan et al., 2016). An explanation for their binding to Aβ could be to stabilize β-helical sheet structure formation.

### LPS in AD Inflammatory Cascades

The prokaryotic functional amyloids and proteolytic enzymes have the ability to modulate and induce host responses, potentially playing a significant role in AD pathogenesis. For example, *P. gingivalis* gingipains also affect cellular functions related to immune signaling. The role of *P. gingivalis* negating the adaptive immune system relates to suppression of interleukin (IL-2) cytokine secretion (Olsen et al., 2016). The lack of IL-2 enhances innate and humoral immune responses resulting in a different cytokine profile. This changes the T helper 17 (Th17) cell lineage, in the modulation of the Th17/T-regulatory cell (Treg) clone formation. The result is an imbalance in Th17 and Treg populations as discussed elsewhere (Olsen et al., 2016). The predominant signaling cascades participating in the innate immune system in AD pathogenesis as mentioned earlier include CD14, TLRs and the NF-κB pathways (Lukiw, 2016), the cAMP-signaling pathway, the transformation growth factor-beta signaling pathway (TGF-β) and the p38 mitogen-activated protein kinase signaling (p38 MAPK) pathway. The latter signaling cascade mediates inflammatory and stress responses and is critical in regulating levels of multiple pro-inflammatory cytokines, such as tumor necrosis factor-α (TNF-α), IL-1, IL-6 and IL-8, as well as enzymes involved in inflammatory cascades e.g., cyclooxygenase and inducible nitric oxide synthase (Chen et al., 1999; Underwood et al., 2000; Huang et al., 2004). TNF-α cytokine is significantly upregulated in AD (Tarkowski et al., 1999), which could be the result of host’s intrinsic genetic factors such as presenilin 2 and APOE4 allele inheritance (Jayadev et al., 2013; Fan et al., 2017), whilst co-morbidities from periodontitis and its etiological polymicrobial pathogens are also responsible for contributing to this cytokine pool (Kamer et al., 2009). A consequence of high levels of TNF-α is that, together with its converting enzyme, this cytokine can provide positive feedback between the γ-secretase site fragmentations of the APP into amyloid-alpha (Aα; Allen, 2017). Infections also induce oxidative stress and this too can have an impact on Aα and Aβ levels through the γ- and β-secretase cleavage of APP (Tamagno et al., 2008). Some GI tract microbiome bacteria can alter γ-aminobutyric acid neurotransmitter signaling by metabolizing glutamate and this has direct implications for impaired cognition as described by Pistollato et al. (2016).

## Future Perspectives

From periodontal microbiome perspectives, and rightly so, much research has focused on making periodontitis an accepted modifiable risk factor, for AD. The near future should recapitulate the AD hallmark protein formation with periodontal polymicrobial oral infections in AD transgenic models. The foreign amyloid-like proteins should be isolated and tested for their true potential to contribute to inflammation and protein-protein interactions leading to senile plaques in AD. The results will open up tantalizing modifiable therapies whereby clinicians may treat or prevent a range of chronic inflammatory diseases orally through dental intervention, diet (probiotics) and education.

## Conclusion

Undoubtedly, a complex etiology underlies the clinical manifestations seen in AD. Candidate microbes conforming to the AD microbiome would be those that induce immunosuppression, are pathogenic, are able to evade the innate and adaptive immune recognition, incite local inflammation and are incapable of allowing entry of activated peripheral blood myeloid cells in the brain. The periodontal microbiome does concur with the type of expected bacteria in AD brains. As an analogy to the dysbiotic periodontal microbial communities driving periodontal disease, the AD microbiome may reflect similar traits. One such example is the keystone periodontal pathogen *P. gingivalis*, which is a master immune evader and an immunosuppressor of the host through IL-2 suppression. Although *P. gingivalis* lacks the curli gene, it has alternative inflammatory mechanisms to indirectly activate β secretases and contribute to host derived Aβ as well as correlate with loss of mental function. A recent systematic review and a 16-year follow-up retrospective cohort study significantly link 10-year exposure to chronic periodontitis as a risk factor for AD. These reports, together with effort from other researchers firmly places periodontitis as a risk factor for AD. Alzheimer’s Research UK charity, suggests that one third of all AD cases are preventable by reducing modifiable risk factors. This is equivalent to at least 198,000 people in the UK unnecessarily suffering from an untreatable, mental illness. With periodontitis and AD showing significant associations, preventative measures must include dental care as an intervention for all members of the society from an early age.

## Author Contributions

ABP initiated the review and wrote most of it. SKS and IO reviewed and corrected the finer details as PhD supervisors of ABP. SC provided critical feedback and funding.

## Conflict of Interest Statement

The authors declare that the research was conducted in the absence of any commercial or financial relationships that could be construed as a potential conflict of interest.

## Footnotes

^1^http://www.hmpdacc.org/

^2^www.homd.org

^3^http://www.hmpdacc.org/

^4^www.homd.org
